# Editorial: Explainable AI in Natural Language Processing

**DOI:** 10.3389/frai.2024.1472086

**Published:** 2024-08-16

**Authors:** Somnath Banerjee, David Tomás

**Affiliations:** ^1^Institute of Computer Science, University of Tartu, Tartu, Estonia; ^2^Department of Software and Computing Systems, University of Alicante, San Vicente del Raspeig, Spain

**Keywords:** explainability, interpretability, rationalization, artificial intelligence, deep learning

The rapid advancement of artificial intelligence (AI) in recent years, and more specifically deep learning techniques, has significantly enhanced the performance of Natural Language Processing (NLP) tasks, ranging from sentiment analysis to image-to-text models. Despite these advancements, the “black box” nature of deep learning models presents a critical challenge: low interpretability. This lack of transparency hinders the application of these models in crucial domains where understanding the decision-making process is essential. This is the case, for example, of healthcare systems, where professionals must understand the rationale behind the model recommendations to ensure they are accurate, safe, and tailored to the patient's specific needs.

Without explainability, there is a risk of undetected biases, errors, or incorrect assumptions within the models. In this editorial, we explore the latest research efforts aimed at making AI models in NLP more explainable and interpretable. The following is a description of each of the five papers accepted in this Research Topic.

The study by Quan et al. is an attempt to develop trustworthy sentiment analysis systems by combining attention-based analysis and the integration of external knowledge. In their approach, the authors propose model training using a multi-task learning approach, augmenting with an attention mechanism to assign scores to evidence. This multi-task approach reduces bias and provides a more comprehensive understanding of the underlying sentiment. Furthermore, an external knowledge base is employed to retrieve complete evidence phrases, thus enhancing the prediction rationality and loyalty of the model (evidence extraction).

Usually, in multimodal settings, such as those involving the integration of vision and language (e.g., image captioning), the explainability of models is provided by token-by-token, which is hard to interpret as they are token-specific. Some explainability methods, such as SHAP (SHapley Additive exPlanations), reduce interpretability by considering superpixels as features which do not correspond to semantically meaningful regions of an image. To mitigate these issues of current approaches, Cafagna et al. propose a SHAP-based framework that generates sentence-based semantically more expressive explanations than traditional methods at a lower compute cost. Also, it reduces the number of visual input features by exploiting the semantics embedded in the models' visual backbone, which yields computation costs.

There has recently been growing interest in developing probing approaches for Pretrained Language Models (PLM) based on Construction Grammar (CxG), that emphasizes the connection between syntax and semantics. The study by Weissweiler et al. examines how well PLMs can recognize and understand a CxG construction, specifically the English Comparative Correlative (CC), which is one of the most commonly studied constructions in linguistics. The authors propose a way of testing the PLMs' recognition of the CC that overcomes the challenge of probing for linguistic phenomena not lending themselves to minimal pairs. In their experiments, they employ BERT, RoBERTa, and DeBERTa as pretrained language models for their understanding of the CC in zero-shot settings. This study observed that PLMs are able to recognize the structure of the CC but fail to use its meaning in diverse NLP tasks.

Nowadays, deep learning based NLP applications also influence the lives of non-experts. This is the case, for example, of a bank system with an NLP model that automatically denies a loan application. Therefore, non-experts also deserve to know and understand how these black-box systems work on the inside. In these scenarios, rationalization emerges as a more accessible explainable technique in NLP. Rationalization justifies a model's output by providing a natural language explanation (rationale). The survey by Gurrapu et al. presents available methods, explainable evaluations, code, and datasets used across various NLP tasks that use rationalization.

The survey developed by Herrewijnen et al. is also in line with the human friendliness of an explanation for non-technical users. This work aims to provide insight into the lessons learned in collecting and using annotator rationales in NLP. To this end, the authors surveyed the use of annotator rationales in the field of NLP, specifically for explainable text classification. As previous studies, they recommend that the scientific community would benefit from the construction of new datasets containing human-annotated rationales as well as task specific investigation of human-annotated rationales that can aid data collection and model training.

The works presented in this editorial highlight different approaches aimed at improving the explainability and interpretability of AI models in NLP. One common theme among the studies is the integration of additional layers of analysis and knowledge to explain model decisions. For instance, combining attention-based mechanisms with external knowledge sources can provide more comprehensive insights into model behavior, thus enhancing interpretability. These methods help reduce bias and improve the completeness of evidence extraction, making the models more trustworthy and their decisions more understandable.

Another significant approach discussed is the use of frameworks that generate semantically rich explanations. The concept of rationalization emerges as a powerful tool for making AI decisions understandable to non-experts. Providing natural language explanations for model outputs bridges the gap between technical complexity and human comprehensibility. This approach is particularly beneficial in applications where the end-users are not technically trained, ensuring that they can understand and trust the AI's decisions.

The collective findings of these research efforts underscore the importance of explainability in the development and deployment of AI systems. By focusing on creating models that are not only accurate but also transparent and interpretable, it can ensured that AI technologies are both effective and trustworthy. The pursuit of explainable AI is crucial for fostering broader social acceptance and ethical implementation of AI systems across many different domains, ultimately leading to more robust and reliable technological advancements in this area.

